# Supplementary material for: Galápagos yellow warblers differ in behavioural plasticity in response to traffic noise depending on proximity to road
Source: Anim Behav. Author manuscript; Available in PMC 2026 May 20. (PMC7619090; doi:10.1016/j.anbehav.2025.123119)
Supplement: Suplementary [file EMS213481-supplement-Suplementary.zip › 1-s2.0-S0003347225000466-mmc1.docx]

**Supplementary Materials**

**Table S1**

Factor loadings for the aggression scores (PC1)

|  | Loading coefficients |
| --- | --- |
| Closest approach | −0.77 |
| No. of crosses over speaker | 0.91 |
| No. of flights | 0.93 |

**Table S2**

Changes in behaviour in response to conspecific playback

| Preplayback behaviour | | | | | Playback behaviour | | | |
| --- | --- | --- | --- | --- | --- | --- | --- | --- |
|  | Floreana | | Santa Cruz | | Floreana | | Santa Cruz | |
|  | Song | Song + noise | Song | Song + noise | Song | Song + noise | Song | Song + noise |
| No. of songs | 2 ± 0.4 | 2 ± 0.4 | 3.3 ± 0.6 | 4.3 ± 0.7 | 2.9 ± 0.4 | 2.8 ± 0.3 | 3.7 ± 0.5 | 3.8 ± 0.5 |
| No. of crosses | 0 | 0.1 ± 0.1 | 0.1 ± 0.1 | 0 | 1.3 ± 0.3 | 1.4 ± 0.2 | 1.4 ± 0.3 | 1.3 ± 0.4 |
| No. of flights | 1.4 ± 0.4 | 0.7 ± 0.2 | 1.2 ± 0.4 | 0.9 ± 0.3 | 2.7 ± 0.3 | 2.9 ± 0.4 | 2.5 ± 0.4 | 2.5 ± 0.5 |

The behaviour shown as number of songs and movement events per minute (mean ± SE) for yellow warbler males during (1) the 1 min period before the broadcast of the playback stimuli; and (2) during broadcast of either the song or song + noise treatments. On both islands, resident males increased movement in response to the broadcast of song of a simulated intruder with or without noise.

**Table S3**

Outputs of the model-averaged linear mixed models for the number of songs produced during playbacks based on all the top models with ∆corrected Akaike information criterion ≤6

| Fixed effects | Estimate | SE | 95% CI | Relative variable importance |
| --- | --- | --- | --- | --- |
| (Intercept) | 7.88 | 1.35 | (5.23, 10.53) |  |
| islandSanta Cruz | 4.78 | 2.46 | (−0.04, 9.60) | 0.95 |
| territory_locationroadside | −0.26 | 1.64 | (− 3.47, 2.94) | 0.83 |
| treatment_ordersecond trial | 1.74 | 1.48 | (−1.17, 4.64) | 0.73 |
| islandSanta Cruz:territory_locationroadside | −3.59 | 3.29 | (−10.05, 2.87) | 0.65 |
| treatmentSong + Noise | −0.08 | 0.71 | (−1.47, 1.30) | 0.24 |
| territory_locationroadside:treatmentSong + Noise | 0.06 | 0.56 | (−1.03, 1.15) | 0.03 |
| islandSanta Cruz:treatmentSong + Noise | 0.03 | 0.44 | (−0.84, 0.90) | 0.03 |

All models included bird ID as the random factor.

**Table S4**

Outputs of the model-averaged linear mixed models for the maximum frequencies of songs based on all the top models with ∆corrected Akaike information criterion ≤6

| Fixed effects | Estimate | SE | 95% CI | Relative variable importance |
| --- | --- | --- | --- | --- |
| (Intercept) | 7127.51 | 112.94 | (6906.16, 7348.86) |  |
| treatmentSong + Noise | 76.30 | 76.78 | (–74.18, 226.79) | 0.72 |
| treatment_ordersecond trial | 26.08 | 50.58 | (–73.06, 125.22) | 0.39 |
| territory_locationroadside | –24.38 | 124.32 | (–268.03, 219.28) | 0.41 |
| islandSanta Cruz | –6.89 | 138.56 | (–278.46, 264.67) | 0.40 |
| islandSanta Cruz:treatmentSong + Noise | 11.47 | 51.54 | (–89.54, 112.48) | 0.10 |
| territory_locationroadside:treatmentSong + Noise | –2.17 | 31.98 | (–64.85, 60.51) | 0.06 |
| islandSanta Cruz:territory_locationroadside | –42.36 | 163.69 | (–363.17, 278.46) | 0.09 |

All models included bird ID as the random factor.

**Table S5**

Outputs of the model-averaged linear mixed models for the bandwidth of songs based on all the top models with ∆corrected Akaike information criterion ≤6

| Fixed effects | Estimate | SE | 95% CI | Relative variable importance |
| --- | --- | --- | --- | --- |
| (Intercept) | 4646.02 | 117.48 | (4415.76, 4876.28) |  |
| islandSanta Cruz | −70.24 | 167.81 | (–399.13, 258.66) | 0.52 |
| treatmentSong + Noise | 8.82 | 57.01 | (–102.93, 120.56) | 0.48 |
| territory_locationroadside | −29.31 | 128.79 | (–281.72, 223.11) | 0.41 |
| islandSanta Cruz:treatmentSong + Noise | 34.40 | 88.73 | (–139.50, 208.31) | 0.18 |
| treatment_ordersecond trial | 1.75 | 29.71 | (–56.49, 59.98) | 0.25 |
| islandSanta Cruz:territory_locationroadside | −41.96 | 162.71 | (–360.87, 276.96) | 0.10 |
| territory_locationroadside:treatmentSong + Noise | 0.42 | 24.41 | (–47.41, 48.26) | 0.04 |

All models included bird ID as the random factor.

**Table S6**

Model averaging table for the linear mixed models for aggression score as the output of dredge function in R

| isl | loc | trt | ord | Isl:loc | Isl:trt | loc:trt | Isl:loc:trt | *df* | logLik | AICc | Delta | Weight |
| --- | --- | --- | --- | --- | --- | --- | --- | --- | --- | --- | --- | --- |
| + | + | + | + |  | + | + |  | 9 | −84.56805 | 189.8634 | 0.000000 | 0.5569121 |
| + | + | + | + | + | + | + |  | 10 | −84.14828 | 191.6812 | 1.817793 | 0.2244182 |
| + | + | + | + | + | + | + | + | 11 | −83.57123 | 193.2675 | 3.404087 | 0.1015310 |
|  | + | + | + |  |  | + |  | 7 | −89.09211 | 193.8313 | 3.967910 | 0.0765889 |

isl: island, loc: territory location, trt: treatment, ord: treatment order. + indicates that the term was included in that model. Only the models with delta corrected Akaike information criterion (AICc) <6 are shown in the table. Intercept and bird ID as the random effect was included in each model. Intraclass coefficient for the random effect was 0.72 in the top model.

**Table S7**

Model averaging table for the linear mixed models for number of songs produced during the playback as the output of dredge function in R

| isl | loc | trt | ord | Isl:loc | Isl:trt | loc:trt | Isl:loc:trt | *df* | logLik | AICc | Delta | Weight |
| --- | --- | --- | --- | --- | --- | --- | --- | --- | --- | --- | --- | --- |
| + | + |  | + | + |  |  |  | 7 | −231.8795 | 479.4061 | 0.000000 | 0.26892327 |
| + | + |  |  | + |  |  |  | 6 | −233.8639 | 480.9452 | 1.539063 | 0.12457336 |
| + | + | + | + | + |  |  |  | 8 | −231.8681 | 481.8854 | 2.479300 | 0.07784939 |
| + | + |  | + |  |  |  |  | 6 | −234.3588 | 481.9350 | 2.528832 | 0.07594504 |
| + |  |  | + |  |  |  |  | 5 | −235.7046 | 482.2664 | 2.860240 | 0.06434802 |
| + | + |  |  |  |  |  |  | 5 | −236.2208 | 483.2988 | 3.892686 | 0.03840099 |
| + | + | + |  | + |  |  |  | 7 | −233.8630 | 483.3730 | 3.966821 | 0.03700362 |
| + |  |  |  |  |  |  |  | 4 | −237.5122 | 483.5877 | 4.181543 | 0.03323670 |
| + | + | + | + | + |  | + |  | 9 | −231.5866 | 483.9005 | 4.494400 | 0.02842378 |
|  |  |  | + |  |  |  |  | 4 | −237.7742 | 484.1117 | 4.705553 | 0.02557588 |
| + | + | + | + | + | + |  |  | 9 | −231.7706 | 484.2685 | 4.862370 | 0.02364711 |
| + | + | + | + |  |  |  |  | 7 | −234.3481 | 484.3432 | 4.937051 | 0.02278040 |
| + |  | + | + |  |  |  |  | 6 | −235.6940 | 484.6055 | 5.199323 | 0.01998066 |
|  | + |  | + |  |  |  |  | 5 | −237.0737 | 485.0045 | 5.598337 | 0.01636685 |

isl: island, loc: territory location, trt: treatment, ord: treatment order. + indicates that the term was included in that model. Only the models with delta corrected Akaike information criterion (AICc) <6 are shown in the table. Intercept and bird ID as the random effect was included in each model. Intraclass coefficient for the random effect was 0 in the top model as bird ID did not account for any variance.

**Table S8**

Model averaging table for the linear mixed models for the minimum frequencies of songs as the output of dredge function in R

| isl | loc | trt | ord | Isl:loc | Isl:trt | loc:trt | Isl:loc:trt | *df* | logLik | AICc | Delta | Weight |
| --- | --- | --- | --- | --- | --- | --- | --- | --- | --- | --- | --- | --- |
| + |  | + | + |  | + |  |  | 7 | –3317.716 | 6649.663 | 0.000000 | 0.29388199 |
| + |  | + | + |  |  |  |  | 6 | –3319.535 | 6651.243 | 1.579652 | 0.13340001 |
| + | + | + | + |  | + |  |  | 8 | –3317.713 | 6651.725 | 2.061707 | 0.10482841 |
|  |  | + | + |  |  |  |  | 5 | –3321.000 | 6652.124 | 2.460655 | 0.08587141 |
| + | + | + | + |  | + | + |  | 9 | –3317.454 | 6653.283 | 3.619336 | 0.04811097 |
| + | + | + | + |  |  |  |  | 7 | –3319.535 | 6653.301 | 3.638068 | 0.04766246 |
| + | + | + | + | + | + |  |  | 9 | –3317.666 | 6653.706 | 4.043001 | 0.03892659 |
| + | + | + | + | + | + | + | + | 11 | –3315.587 | 6653.725 | 4.062120 | 0.03855625 |
|  | + | + | + |  |  |  |  | 6 | –3320.925 | 6654.025 | 4.361292 | 0.03319942 |
| + | + | + | + |  |  | + |  | 8 | –3318.924 | 6654.147 | 4.483475 | 0.03123192 |
|  | + | + | + |  |  | + |  | 7 | –3320.221 | 6654.674 | 5.010689 | 0.02399472 |
| + | + | + | + | + | + | + |  | 10 | –3317.418 | 6655.296 | 5.632656 | 0.01758155 |
| + | + | + | + | + |  |  |  | 8 | –3319.513 | 6655.325 | 5.661962 | 0.01732581 |

isl: island, loc: territory location, trt: treatment, ord: treatment order. + indicates that the term was included in that model. Only the models with delta corrected Akaike information criterion (AICc) <6 are shown in the table. Intercept and bird ID as the random effect was included in each model. Intraclass coefficient for the random effect was 0.32 in the top model.

**Table S9**

Model averaging table for the linear mixed models for the maximum frequencies of songs as the output of dredge function in R

| isl | loc | trt | ord | Isl:loc | Isl:trt | loc:trt | Isl:loc:trt | *df* | logLik | AICc | Delta | Weight |
| --- | --- | --- | --- | --- | --- | --- | --- | --- | --- | --- | --- | --- |
|  |  | + |  |  |  |  |  | 4 | –3879.345 | 7766.773 | 0.000000 | 0.155356008 |
|  |  | + | + |  |  |  |  | 5 | –3878.887 | 7767.898 | 1.125594 | 0.088492918 |
|  | + | + |  |  |  |  |  | 5 | –3879.126 | 7768.375 | 1.602633 | 0.069714104 |
|  |  |  |  |  |  |  |  | 3 | –3881.233 | 7768.515 | 1.742262 | 0.065013082 |
| + |  | + |  |  |  |  |  | 5 | –3879.236 | 7768.596 | 1.823736 | 0.062417855 |
|  |  |  | + |  |  |  |  | 4 | –3880.357 | 7768.797 | 2.024368 | 0.056460150 |
|  | + | + | + |  |  |  |  | 6 | –3878.644 | 7769.463 | 2.689902 | 0.040478386 |
| + |  | + | + |  |  |  |  | 6 | –3878.772 | 7769.718 | 2.945794 | 0.035616969 |
| + |  | + |  |  | + |  |  | 6 | –3878.808 | 7769.789 | 3.016416 | 0.034381251 |
|  | + |  |  |  |  |  |  | 4 | –3880.964 | 7770.011 | 3.238087 | 0.030774178 |
|  | + |  | + |  |  |  |  | 5 | –3880.062 | 7770.248 | 3.474982 | 0.027336650 |
|  | + | + |  |  |  | + |  | 6 | –3879.065 | 7770.304 | 3.531576 | 0.026573944 |
| + | + | + |  |  |  |  |  | 6 | –3879.072 | 7770.319 | 3.545961 | 0.026383505 |
| + |  |  |  |  |  |  |  | 4 | –3881.136 | 7770.354 | 3.581437 | 0.025919629 |
| + | + | + |  | + |  |  |  | 7 | –3878.129 | 7770.490 | 3.717425 | 0.024215833 |
| + |  |  | + |  |  |  |  | 5 | –3880.251 | 7770.626 | 3.853627 | 0.022621614 |
| + |  | + | + |  | + |  |  | 7 | –3878.268 | 7770.768 | 3.995206 | 0.021075609 |
| + | + | + | + |  |  |  |  | 7 | –3878.589 | 7771.411 | 4.638096 | 0.015281930 |
| + | + | + | + | + |  |  |  | 8 | –3877.596 | 7771.491 | 4.718130 | 0.014682468 |
|  | + | + | + |  |  | + |  | 7 | –3878.639 | 7771.511 | 4.738129 | 0.014536386 |
| + | + | + |  |  | + |  |  | 7 | –3878.663 | 7771.559 | 4.786523 | 0.014188868 |
| + | + | + |  | + | + |  |  | 8 | –3877.788 | 7771.875 | 5.102041 | 0.012118086 |
| + | + |  |  | + |  |  |  | 6 | –3879.881 | 7771.937 | 5.163855 | 0.011749278 |
| + | + |  |  |  |  |  |  | 5 | –3880.924 | 7771.972 | 5.199054 | 0.011544307 |
| + | + |  | + | + |  |  |  | 7 | –3878.922 | 7772.077 | 5.303929 | 0.010954551 |
| + | + |  | + |  |  |  |  | 6 | –3880.018 | 7772.209 | 5.436715 | 0.010250862 |
| + | + | + |  |  |  | + |  | 7 | –3879.007 | 7772.246 | 5.472859 | 0.010067271 |
| + | + | + |  | + |  | + |  | 8 | –3878.081 | 7772.461 | 5.688650 | 0.009037605 |
| + | + | + | + |  | + |  |  | 8 | –3878.106 | 7772.511 | 5.738233 | 0.008816306 |
| + | + | + | + | + | + |  |  | 9 | –3877.185 | 7772.744 | 5.971708 | 0.007844912 |

isl: island, loc: territory location, trt: treatment, ord: treatment order. + indicates that the term was included in that model. Only the models with delta corrected Akaike information criterion (AICc) <6 are shown in the table. Intercept and bird ID as the random effect was included in each model. Intraclass coefficient for the random effect was 0.32 in the top model.

**Table S10**

Model averaging table for the linear mixed models for the bandwidth frequencies of songs as the output of dredge function in R

| isl | loc | trt | ord | Isl:loc | Isl:trt | loc:trt | Isl:loc:trt | *df* | logLik | AICc | Delta | Weight |
| --- | --- | --- | --- | --- | --- | --- | --- | --- | --- | --- | --- | --- |
|  |  |  |  |  |  |  |  | 3 | –3864.974 | 7735.997 | 0.000000 | 0.142495187 |
| + |  |  |  |  |  |  |  | 4 | –3864.477 | 7737.037 | 1.040541 | 0.084693413 |
|  |  | + |  |  |  |  |  | 4 | –3864.564 | 7737.210 | 1.212700 | 0.077708009 |
|  | + |  |  |  |  |  |  | 4 | –3864.623 | 7737.329 | 1.331844 | 0.073213953 |
| + |  | + |  |  | + |  |  | 6 | –3862.667 | 7737.508 | 1.511505 | 0.066923870 |
|  |  |  | + |  |  |  |  | 4 | –3864.961 | 7738.004 | 2.006795 | 0.052243263 |
| + |  | + |  |  |  |  |  | 5 | –3864.052 | 7738.228 | 2.231677 | 0.046687185 |
|  | + | + |  |  |  |  |  | 5 | –3864.238 | 7738.600 | 2.603219 | 0.038772011 |
| + | + |  |  |  |  |  |  | 5 | –3864.276 | 7738.676 | 2.679269 | 0.037325389 |
| + | + |  |  | + |  |  |  | 6 | –3863.391 | 7738.956 | 2.959656 | 0.032442861 |
| + |  |  | + |  |  |  |  | 5 | –3864.462 | 7739.048 | 3.051199 | 0.030991370 |
|  |  | + | + |  |  |  |  | 5 | –3864.563 | 7739.251 | 3.253767 | 0.028006171 |
| + | + | + |  |  | + |  |  | 7 | –3862.523 | 7739.278 | 3.280781 | 0.027630435 |
|  | + |  | + |  |  |  |  | 5 | –3864.606 | 7739.337 | 3.339919 | 0.026825391 |
| + |  | + | + |  | + |  |  | 7 | –3862.660 | 7739.552 | 3.555634 | 0.024082645 |
| + | + | + |  | + | + |  |  | 8 | –3861.792 | 7739.884 | 3.886844 | 0.020407167 |
| + | + | + |  |  |  |  |  | 6 | –3863.873 | 7739.919 | 3.922588 | 0.020045687 |
| + |  | + | + |  |  |  |  | 6 | –3864.052 | 7740.278 | 4.281526 | 0.016752461 |
| + | + | + |  | + |  |  |  | 7 | –3863.027 | 7740.287 | 4.290123 | 0.016680610 |
|  | + | + |  |  |  | + |  | 6 | –3864.202 | 7740.578 | 4.581449 | 0.014419530 |
|  | + | + | + |  |  |  |  | 6 | –3864.238 | 7740.650 | 4.653134 | 0.013911856 |
| + | + |  | + |  |  |  |  | 6 | –3864.258 | 7740.690 | 4.693110 | 0.013636541 |
| + | + |  | + | + |  |  |  | 7 | –3863.366 | 7740.965 | 4.967889 | 0.011886027 |
| + | + | + |  |  | + | + |  | 8 | –3862.513 | 7741.324 | 5.327626 | 0.009929350 |
| + | + | + | + |  | + |  |  | 8 | –3862.513 | 7741.326 | 5.329505 | 0.009920029 |
| + | + | + |  |  |  | + |  | 7 | –3863.846 | 7741.924 | 5.927215 | 0.007357355 |
| + | + | + | + | + | + |  |  | 9 | –3861.778 | 7741.930 | 5.933268 | 0.007335122 |
| + | + | + |  | + | + | + |  | 9 | –3861.789 | 7741.952 | 5.955215 | 0.007255073 |
| + | + | + | + |  |  |  |  | 7 | –3863.873 | 7741.978 | 5.980959 | 0.007162282 |

isl: island, loc: territory location, trt: treatment, ord: treatment order. + indicates that the fixed factor or interaction term was included that model. Only the models with delta corrected Akaike information criterion (AICc) <6 are shown in the table. Intercept and bird ID as the random effect was included in each model. Intraclass coefficient for the random effect was 0.35 in the top model.

**Table S11**

Model averaging table for the linear mixed models for the peak frequencies of songs as the output of dredge function in R

| isl | loc | trt | ord | Isl:loc | Isl:trt | loc:trt | Isl:loc:trt | *df* | logLik | AICc | Delta | Weight |
| --- | --- | --- | --- | --- | --- | --- | --- | --- | --- | --- | --- | --- |
| + |  | + | + |  | + |  |  | 7 | –3317.716 | 6649.663 | 0.000000 | 0.29388199 |
| + |  | + | + |  |  |  |  | 6 | –3319.535 | 6651.243 | 1.579652 | 0.13340001 |
| + | + | + | + |  | + |  |  | 8 | –3317.713 | 6651.725 | 2.061707 | 0.10482841 |
|  |  | + | + |  |  |  |  | 5 | –3321.000 | 6652.124 | 2.460655 | 0.08587141 |
| + | + | + | + |  | + | + |  | 9 | –3317.454 | 6653.283 | 3.619336 | 0.04811097 |
| + | + | + | + |  |  |  |  | 7 | –3319.535 | 6653.301 | 3.638068 | 0.04766246 |
| + | + | + | + | + | + |  |  | 9 | –3317.666 | 6653.706 | 4.043001 | 0.03892659 |
| + | + | + | + | + | + | + | + | 11 | –3315.587 | 6653.725 | 4.062120 | 0.03855625 |
|  | + | + | + |  |  |  |  | 6 | –3320.925 | 6654.025 | 4.361292 | 0.03319942 |
| + | + | + | + |  |  | + |  | 8 | –3318.924 | 6654.147 | 4.483475 | 0.03123192 |
|  | + | + | + |  |  | + |  | 7 | –3320.221 | 6654.674 | 5.010689 | 0.02399472 |
| + | + | + | + | + | + | + |  | 10 | –3317.418 | 6655.296 | 5.632656 | 0.01758155 |

isl: island, loc: territory location, trt: treatment, ord: treatment order. + indicates that the term was included in that model. Only the models with delta corrected Akaike information criterion (AICc) <6 are shown in the table. Intercept and bird ID as the random effect was included in each model. Intraclass coefficient for the random effect was 0.15 in the top model.

**Table S12**

Model averaging table for the linear mixed models for the duration of songs as the output of dredge function in R

| isl | loc | trt | ord | Isl:loc | Isl:trt | loc:trt | Isl:loc:trt | *df* | logLik | AICc | Delta | Weight |
| --- | --- | --- | --- | --- | --- | --- | --- | --- | --- | --- | --- | --- |
| + |  | + |  |  | + |  |  | 6 | 2.665056 | 6.843801 | 0.000000 | 0.33149703 |
| + |  | + | + |  | + |  |  | 7 | 2.905835 | 8.420695 | 1.576894 | 0.15068204 |
| + | + | + |  |  | + |  |  | 7 | 2.684664 | 8.863037 | 2.019236 | 0.12078364 |
| + | + | + |  |  | + | + |  | 8 | 3.403725 | 9.491925 | 2.648125 | 0.08819555 |
| + | + | + |  | + | + |  |  | 8 | 2.974855 | 10.349666 | 3.505865 | 0.05743686 |
| + | + | + | + |  | + |  |  | 8 | 2.921396 | 10.456585 | 3.612784 | 0.05444695 |
| + | + | + |  | + | + | + |  | 9 | 3.742215 | 10.890569 | 4.046769 | 0.04382632 |
| + | + | + | + |  | + | + |  | 9 | 3.486631 | 11.401738 | 4.557937 | 0.03394189 |
| + | + | + |  | + | + | + | + | 10 | 4.308127 | 11.843037 | 4.999236 | 0.02722133 |
| + | + | + | + | + | + |  |  | 9 | 3.195476 | 11.984049 | 5.140248 | 0.02536816 |
| + | + | + | + | + | + | + |  | 10 | 3.810802 | 12.837687 | 5.993886 | 0.01655479 |

isl: island, loc: territory location, trt: treatment, ord: treatment order. + indicates that the term was included in that model. Only the models with delta corrected Akaike information criterion (AICc) <6 are shown in the table. Intercept and bird ID as the random effect was included in each model. Intraclass coefficient for the random effect was 0.31 in the top model.
